# Infant feeding experiences among teen mothers in North Carolina: Findings from a mixed-methods study

**DOI:** 10.1186/1746-4358-6-14

**Published:** 2011-09-28

**Authors:** Christine M Tucker, Ellen K Wilson, Ghazaleh Samandari

**Affiliations:** 1 Department of Maternal and Child Health, Gillings School of Global Public Health, The University of North Carolina at Chapel Hill, NC, USA; 2 RTI International (a trade name of Research Triangle Institute), Research Triangle Park, NC, USA

**Keywords:** Breastfeeding, adolescents, barriers, mixed methods

## Abstract

**Background:**

Adolescent mothers in the U.S. are much less likely to initiate breastfeeding than older mothers, and teens who do initiate breastfeeding tend to breastfeed for shorter durations. The purpose of this mixed-methods study is to investigate breastfeeding practices, barriers and facilitators among adolescent mothers ages 17 and younger.

**Methods:**

Quantitative descriptive analyses are conducted using data from the North Carolina Pregnancy Risk Assessment Monitoring System (PRAMS). The population-based sample comprises 389 teens ages 13-17 giving birth to a live born infant in North Carolina in 2000 - 2005 and in 2007. Qualitative analyses are based on in-depth interviews with 22 Black, White and Hispanic teen mothers residing in rural and urban areas of North Carolina conducted between November 2007 and February 2009.

**Results:**

In quantitative analyses, 52% (196 of 389) of North Carolina teen mothers initiated breastfeeding, but half of those who initiated breastfeeding (92/196) stopped within the first month postpartum. Hispanic teens (44/52 or 89%) were much more likely than Black (61/159 or 41%) or White teens (87/164 or 52%) to initiate breastfeeding and to continue for a longer duration. Nearly sixty two percent (29/52) of Hispanic respondents breastfed for greater than four weeks as compared to 16% (29/159) of Black respondents and 26% (39/164) of White respondents. Common barriers to breastfeeding initiation and continuation included not liking breastfeeding, returning to school, nipple pain, and insufficient milk. Qualitative data provided context for the quantitative findings, elucidating the barriers and facilitators to breastfeeding from the teens' perspective and insight into the ways in which breastfeeding support to teens could be enhanced.

**Conclusions:**

The large number of adolescents ceasing breastfeeding within the first month points to the need for more individualized follow-up after hospital discharge in the first few days postpartum, to address common technical challenges and to provide assistance managing the transition back to school. Provision of an extra home visit or outpatient visit for teens within the first few days following hospital discharge, and advocacy to make schools more compatible with breastfeeding, could potentially help teens who desire to breastfeed to successfully continue. These interventions warrant further research to test their effectiveness among adolescents.

## Background

In the United States, just over half of mothers under age 20 initiate breastfeeding [1]. Furthermore, adolescents who do initiate breastfeeding tend to breastfeed for shorter durations than older mothers. According to the National Immunization Survey, between 2004 and 2008 only 19% of teens continued to breastfeed at six months postpartum, compared with 34% of mothers ages 20-29 and 49% of women 30 and older [1].

Several factors may explain the lower levels of breastfeeding among teen mothers. Given their age, teen mothers are more likely than older mothers to be single and to have lower levels of education and income--characteristics that are negatively associated with breastfeeding [2-4]. Additional factors that may contribute to lower breastfeeding rates among teens include returning to school [5], unease with the act of breastfeeding [5], and embarrassment about breastfeeding in public [5,6]. A lack of confidence in their ability to breastfeed [7] and reticence to ask for help may be other barriers that pertain especially to teens [7,8].

Current understanding of breastfeeding among adolescents has several limitations, however. Because national statistics group together mothers younger than 20, we do not have population-based data on the breastfeeding practices specific to younger school-age adolescents. In addition, most studies of adolescent breastfeeding are composed of hospital-based convenience samples of urban White and African American teens [6]. As a result, available data focus primarily on breastfeeding intentions and decisions to initiate, and few studies examine breastfeeding experiences after hospital discharge [8]. Furthermore, there is little information about breastfeeding experiences among Hispanic adolescents or among adolescents from rural areas in the United States.

The purpose of this study is to obtain a better understanding of breastfeeding practices, including the barriers and facilitators to initiation and continuation, among teen mothers. We use both quantitative and qualitative methods to investigate breastfeeding experiences among a racially and ethnically diverse group of young adolescent mothers (ages 13-17) in urban and rural areas of North Carolina. Our mixed-methods design enables us to obtain representative data on breastfeeding outcomes among adolescent mothers and at the same time, explore in depth the contextual factors that promote or inhibit breastfeeding initiation and continuation among a diverse sample of young teens.

## Methods

### Quantitative component

We analyzed six years of data (2000-2005 and 2007) from the North Carolina Pregnancy Risk Assessment Monitoring System (PRAMS). Data from 2006 were excluded because a response rate threshold of 70% was not reached for that year's overall sample (F. Simsek, MPH, North Carolina State Center for Health Statistics, written communication, August 5, 2009). PRAMS is a continuous state-level survey of women who deliver live-born infants. It is designed to collect information on a range of health behaviors and experiences before, during, and after pregnancy. In North Carolina, a stratified systematic sample of approximately 200 new mothers ages 13 or older is drawn each month from a frame of eligible birth certificates, for a total of approximately 2400 mothers per year. Up to three self-administered questionnaires in English or Spanish are mailed to the mothers two to four months after delivery, and telephone interviews are conducted with those who do not respond by mail. Thus, the reference period ranges from two to seven months postpartum. More information about PRAMS is available [9].

Out of 11,567 mothers participating in PRAMS in the years 2000-2005 and 2007, 477 were teens 17 or younger at the time of delivery. Of these, 393 reported that their baby was alive and living with them and thus were eligible for the study. Four adolescents (1%) did not respond to the main infant feeding question, "Did you ever breastfeed or pump breast milk to feed your baby?" and were excluded from the analysis. The final sample comprised 389 adolescents interviewed at a median of four months postpartum. Adolescent mothers participating in PRAMS ranged in age from 13 to 17 with a mean age of 16.3 years. Characteristics of the quantitative sample are summarized in Table 1.

**Table 1:** Demographic characteristics of the quantitative sample in North Carolina (n = 389)

Characteristic	n	(%)
**Age at interview**		
13	1	(0.1)
14	12	(1.8)
15	50	(13.1)
16	126	(30.9)
17	200	(54.2)
**Race/ethnicity**		
African American	164	(45.1)
White	159	(34.0)
Hispanic	52	(17.8)
Other	14	(3.1)
**Marital status**		
Married	40	(11.5)
Not married	349	(88.5)
**Baby's age at interview (months)**		
1-3	163	(44.1)
4-6	217	(55.4)
7-9	2	(0.6)
Missing	7	
**Previous live birth?**		
Yes	30	(8.5)
No	348	(91.5)
Missing	12	
**Trying to get pregnant?**		
Yes	68	(20.8)
No	317	(79.2)
Missing	4	
Delivery paid by...*		
Medicaid	352	(89.9)
Income	13	(3.0)
Insurance/HMO	46	(14.2)
Other	8	(1.7)
**WIC during pregnancy?**		
Yes	325	(85.9)
No	62	(14.1)
Missing	2	
**Main source of prenatal care**		
Hospital clinic	65	(17.3)
Health department clinic	139	(39.9)
Private doctor or HMO clinic	136	(34.2)
Other	32	(8.7)
Missing	17	

*Note*. All data were derived from the North Carolina Pregnancy Risk Assessment Monitoring System (NC PRAMS), 2000-2005 and 2007. The study sample included teens with live births who were 13 to 17 years old at the time of delivery. Teens who did not answer the breastfeeding question were excluded. Data are weighted to account for the sampling, nonresponse, and noncoverage. WIC = Special Supplemental Nutrition Program for Women, Infants, and Children, which provides food and nutrition education for low income pregnant and postpartum women and to infants and children up to age five at nutritional risk. *Women could choose more than one option.

We assessed the prevalence of breastfeeding initiation, duration, and exclusivity, barriers to breastfeeding, and whether a health care worker discussed breastfeeding with teens prenatally. Exclusive breastfeeding was assessed by asking how old the baby was the first time he or she was fed anything besides breast milk. Due to the small number of participants breastfeeding through three months postpartum, we measured exclusive breastfeeding at four weeks postpartum. To assess barriers to breastfeeding initiation, mothers who did not initiate (n = 193) were asked to choose their reasons for not breastfeeding from a list of predetermined responses. These barrier questions were not asked in years 2000-2001 when 78 teens responded to the survey, thus our sample of mothers with information on barriers to initiation was reduced to 115. To assess barriers to breastfeeding continuation, 147 teens who had stopped breastfeeding by the time of the interview were eligible for these questions. Again, none of the barrier questions were asked in years 2000-2001 when 45 teens responded to this question. Additionally, three teens either did not answer the question or responded that they did not know their reasons for cessation, giving a final sample of 102 teens with information on barriers to continuation. For both questions on barriers to initiation and continuation, only response options listed in all survey years of our study are presented here. We also examined reasons for cessation at two breastfeeding duration intervals: less than four weeks and after four weeks.

Using SAS software 9.1.3 and SUDAAN 9 [10] we applied state-specific statistical weights to account for the sampling design, nonresponse, and noncoverage. We computed frequency distributions of each selected breastfeeding outcome by race/ethnicity and used chi-square tests to determine the statistical significance of the association of each indicator with the mother's race/ethnicity. We present unweighted frequencies and weighted percentages to reflect the entire population of North Carolina females ages 13-17 having live births.

### Qualitative component

The qualitative component consisted of semistructured interviews with 22 adolescent mothers in central North Carolina between November 2007 and February 2009. We developed a semistructured interview guide based on key constructs from the theory of planned behavior [11] and from interviews with six key informants who worked closely with adolescent mothers. Following the Theory of Planned Behavior [11], we conceptualized adolescent mothers' health behaviors as being jointly determined by their intention to perform the behaviors and their actual ability to do so. Intention to perform the behavior was in turn determined by their attitude toward the behavior, subjective norm regarding the behavior, and perceived ability to perform the behavior. Thus qualitative participants were asked about their personal attitudes toward breastfeeding including positive and negative aspects, family and friends' norms around breastfeeding, perceived and actual ability to breastfeed including information and support received and barriers encountered, and intentions to breastfeed prior to delivery. Our definition of breastfeeding included exclusive breastfeeding, breastfeeding and supplementing with formula, pumping breast milk, and any combination of these.

To recruit study participants, staff from organizations serving adolescent mothers (adolescent parenting programs, health departments, community health centers, high schools, and general equivalency diploma [GED] programs) distributed flyers describing the study to any adolescents who might be eligible. Because we were particularly interested in the experiences of young, first-time teen mothers, we limited the sample to those who were under 18 years of age at the time they gave birth and prioritized those who had had only one child. We conducted purposive sampling to maximize variation according to urban and rural areas and racial/ethnic group (Black, White, and Hispanic) to ensure that a variety of views and experiences were captured. We continued recruitment until we had achieved a minimum of three participants from each racial/ethnic group from both the rural and urban areas.

Between November 2007 and February 2009, three female interviewers trained in qualitative data collection techniques conducted 22 semistructured interviews in English. Interviews with teens lasted between an hour and an hour and a half and were conducted in a private location (usually the respondent's home). consent and compensated respondents $50 for their time. Interviews were recorded and transcribed verbatim. All procedures were approved by RTI's Institutional Review Board. Characteristics of the qualitative sample are shown in Table 2. At the time they gave birth, the teens ranged in age from 13 to 17.

**Table 2:** Demographic characteristics of the qualitative sample in central North Carolina (n = 22)

Characteristic	Total
**Age at delivery**	
13	1
14	1
15	7
16	6
17	7
**Race/ethnicity**	
White	6
Black	8
Hispanic	8
**Marital status**	
Married	0
Other	22
**Age of the baby at time of interview**	
< 6 months	8
6 months 1 year	11
> 1 year	3
**Previous live birth?**	
Yes	1
No	21
**Trying to get pregnant?**	
Yes	2
No	20
**Delivery paid by..**.	
Medicaid	21
Unsure	1
**Urban/rural residence**	
Urban	13
Rural	9

*Note*. Qualitative sample includes data from in-depth individual interviews with teen mothers in North Carolina conducted between November 2007 and February 2009.

Transcripts were imported into NVivo8 Software for analysis and coded thematically. We developed a preliminary codebook based on the topic domains and themes that emerged in the interviews. After multiple readings of the transcripts, all data coded under the theme of infant feeding were further analyzed using matrices to summarize breastfeeding experiences within the larger context of teens' lives following the example of Bentley et al. in a previous study on infant feeding among adolescents [12]. Matrices were used to further compare text across racial ethnic groups and between rural and urban settings. Memo writing and ongoing discussions with the research team helped to verify the accuracy of the interpretations and to resolve any discrepancies. To provide context, quotes presented in this paper are labeled by urban or rural residence, race/ethnicity, type and duration of infant feeding. Ellipses are used in quotations to indicate where words were omitted to make long quotations more concise. To quantify the number of participants who experienced each statement, in some cases we present exact numbers and in other instances we use the words few, some, and many to connote less than half, about half, and more than half.

## Results

We begin by summarizing the quantitative and qualitative data on infant feeding outcomes, followed by the barriers to breastfeeding and influences on breastfeeding. Throughout the results section, we use the qualitative data to delve more deeply into the quantitative results as well as to explore themes not covered by PRAMS.

### Infant feeding outcomes

Data from PRAMS indicate that just over half (52%) of North Carolina teen mothers age 17 or younger initiated breastfeeding during 2000-2005 and 2007 (Table 3). Due to the small sample size of adolescents identifying as "other" race-ethnicity, we focus racial and ethnic comparisons between White, Black, and Hispanic youth. Nearly all Hispanic teens initiated breastfeeding (89%), compared with about half (52%) of Whites and a third (35%) of Blacks. At one month postpartum, only a quarter of teens continued to breastfeed. Breastfeeding duration was greatest for Hispanic teens: over 60% of Hispanic respondents breastfed for longer than four weeks, compared with 26% of White and 17% of Black adolescents. Many teens who initiated breastfeeding did not breastfeed exclusively. Only 17% of participants breastfed exclusively through four weeks postpartum (Table 3).

**Table 3:** Breastfeeding outcomes by race/ethnicity among adolescents ages 17 and younger in North Carolina's Pregnancy Risk Assessment Monitoring System (n = 389)

	Total		White		Black		Hispanic		Other		
	n = 389	(100%)	n = 164	(42.2%)	n = 159	(40.9%)	n = 52	(13.4%)	n = 14	(3.6%)	p-value*
Ever breastfed**											
Yes	196	(52.1)	87	(52.2)	61	(34.6)	44	(88.7)	4	(34.7)	
No	193	(47.9)	77	(47.8)	98	(65.4)	8	(11.3)	10	(65.3)	0.000
**Breastfeeding duration**											
None	193	(48.5)	77	(48.9)	98	(65.7)	8	(11.4)	10	(65.3)	
< 1 week	13	(4.4)	7	(6.3)	4	(3.1)	0	(0.0)	2	(17.6)	
1 - 4 weeks	79	(18.8)	38	(18.8)	25	(14.7)	14	(26.7)	2	(17.0)	
> 4 weeks	97	(28.4)	39	(26.0)	29	(16.5)	29	(61.9)	0	(0.0)	0.000
Missing	7		3		3		1				
Breastfeeding exclusively through 4 weeks***											
Yes	59	(16.9)	29	(19.3)	17	(8.4)	13	(30.3)	0	(0.0)	
No	322	(83.2)	132	(80.7)	139	(91.6)	37	(69.7)	14	(100.0)	0.003
Missing	8		3		3		2				
**Health care worker discussed breastfeeding**											
Yes	356	(93.7)	154	(96.4)	145	(94.7)	44	(83.8)	13	(100.0)	
No	23	(6.3)	8	(3.6)	9	(5.3)	6	(16.2)	0	(0.0)	0.084
Missing	10		2		5		2		1		

*Note*. All data were derived from the North Carolina Pregnancy Risk Assessment Monitoring System (NC PRAMS), 2000-2005 and 2007. The study sample included teens with live births who were 13 to 17 years old at the time of delivery. Teens who did not answer the breastfeeding question were excluded. Data are weighted to account for the sampling, nonresponse, and noncoverage. *Based on chi-square significance tests. **Refers to teens who ever breastfed or pumped breast milk for baby after delivery. ***Refers to teens who have not fed their baby anything other than breast milk through four weeks postpartum.

In the qualitative study, 17 out of 22 participants initiated breastfeeding (Table 4). Similar to the quantitative findings, the duration of breastfeeding for many of the qualitative participants was short. Only eight of the 17 who initiated breastfeeding breastfed longer than four weeks, and many of these teens supplemented breast milk with formula or used a breast pump to give breast milk.

**Table 4:** Summary of breastfeeding practices, barriers, and influences among adolescent mothers 17 and younger in North Carolina (n = 22)

	Qualitative findings
**Practices**	• Many teens (17 out of 22) initiated breastfeeding
	• Half of those who initiated stopped within the first month, and many supplemented with formula or used the breast pump to give milk
	• Compared with Whites and Hispanics, fewer African Americans initiated breastfeeding, and more discontinued within the first 2 weeks

**Barriers**	**Reasons for not initiating**
	• Fear of pain
	• Anticipation of difficulty upon return to school
	• No clearly articulated reason: "just didn't want to"
	**Reasons for stopping**
	• Pain
	• Difficulty latching on and insufficient breast milk
	• Returning to school-concerns included getting enough sleep, leakage, and difficulty and frequency of pumping

**Influences**	**Influences on initiation**
	• Many teens said healthcare providers encouraged breastfeeding during prenatal care and at delivery
	• Many teens got support and encouragement to breastfeed from family though this was less common among Black teens
	• Having family members who had breastfed motivated some teens to try breastfeeding
	• Negative breastfeeding experiences of peers dissuaded a few teens from breastfeeding
	**Influences on continuation**
	• Few teens received hands-on professional assistance after hospital discharge
	• Encouragement from family did not help teens overcome technical difficulties

*Note*. Qualitative findings are based on data from in-depth individual interviews with teen mothers in North Carolina conducted between November 2007 and February 2009.

### Barriers to breastfeeding

In the quantitative data, of the 115 teens who never breastfeed, about half indicated more than one reason for not breastfeeding (Table 5). The most frequently chosen reasons were not liking breastfeeding in general (59%), returning to school (43%), feeling embarrassed (17%), not wanting to be tied down (14%), and "other" (26%). "Other" reasons included: having twins, not producing enough milk, the baby's refusing to breastfeed, diabetes, and smoking.

**Table 5:** Barriers to breastfeeding among adolescents 17 and younger in North Carolina's Pregnancy Risk Assessment Monitoring System

	Yes	(%)
**Number of reasons given for not initiating breastfeeding (n = 115)**		
0	2	(2)
1	56	(47)
2	33	(28)
3+	24	(24)
Reasons for not initiating breastfeeding*		
I didn't like breastfeeding	66	(59)
I went back to work or school	44	(43)
Other	31	(26)
I was embarrassed to breastfeed	13	(17)
I didn't want to be tied down	15	(14)
I wanted my body back to myself	15	(11)
I had too many household duties	9	(9)
I had other children to take care of	6	(4)
**Number of reasons given for stopping breastfeeding (n = 102)**		
1	40	(37)
2	26	(24)
3+	36	(40)
Reasons for stopping*		
I went back to work or school	30	(34)
Breast milk alone did not satisfy my baby	28	(33)
I thought I was not producing enough milk	39	(32)
My nipples were sore, cracked, or bleeding	26	(28)
Other	28	(19)
My baby had difficulty nursing	22	(19)
I felt it was the right time to stop breastfeeding	13	(16)
I wanted/needed someone else to feed the baby	11	(13)
I thought my baby was not gaining enough weight	12	(10)
I had too many other household duties	12	(9)
I got sick and could not breastfeed	3	(6)
My baby got sick and could not breastfeed	4	(5)

*Note*. All data were derived from the North Carolina Pregnancy Risk Assessment Monitoring System (NC PRAMS), 2000-2005 and 2007. The study sample included teens with live births who were 13 to 17 years old at the time of delivery. Teens who did not answer the breastfeeding question or were not asked questions on barriers with response options for all study years were excluded. 115 participants who did not initiate were asked their reasons for not breastfeeding. 102 participants who had stopped breastfeeding by the time of the survey were asked their reasons for stopping. Data are weighted to account for the sampling, nonresponse, and noncoverage. *Teens could choose more than one reason.

Of the 102 respondents who had initiated breastfeeding but stopped by the time of the survey (median four months postpartum), nearly two thirds listed more than one reason for stopping. The most common reasons for discontinuing breastfeeding included returning to school (34%), feeling that breast milk alone did not satisfy the baby (33%) or that they were not producing enough milk (32%), nipple pain (28%), and "other" reasons (19%; Table 5). "Other" reasons for cessation included: baby was in the hospital, baby did not want it anymore or was not eating enough, baby was teething, teen's milk supply diminished, and teen did not like leaking. Nipple pain was cited as a reason for cessation more often among those who stopped breastfeeding early than it was among those who breastfed for longer periods (21/62 or 40% among those who stopped within four weeks, compared with 5/39 or 12% among those who breastfed longer than four weeks; not shown). No other reasons for cessation differed significantly by the timing of cessation.

In the qualitative study, five of the 22 participants did not initiate breastfeeding (Table 4). Three of these five had considered breastfeeding, but were dissuaded by what they perceived to be the negative experiences of friends or relatives. The other two participants said that they had never considered breastfeeding. Neither of them articulated a clear reason for not wanting to breastfeed, although one rural African American who did not breastfeed said that she did not like the idea of "putting [her] booby in her [baby's] mouth." Two participants also anticipated that it would be too difficult to breastfeed when they returned to school, and in general, they thought that formula seemed simpler.

Of the 17 participants who initiated breastfeeding, 15 had stopped by the time of the interview (between one and 18 months postpartum) and gave multiple reasons for breastfeeding discontinuation which were largely intertwined (Table 4). As described in more detail below, the main reasons were similar to those cited in the PRAMS survey, including painful experiences breastfeeding, difficulty latching on, uncertainty whether the baby was getting enough milk, and the challenge of combining school and breastfeeding. Less common reasons for stopping were that breastfeeding took too long and that they did not like leaking milk.

#### Physical discomfort

Seven of the 17 participants who initiated breastfeeding cited pain as a reason for stopping. Some found breastfeeding to be painful from the beginning and stopped or switched to pumping almost immediately. Others managed to breastfeed or pump for several weeks or months, but developed mastitis or bleeding nipples, which contributed to their decision to stop. As one rural African American teen who fed breast milk for six months and pumped breast milk after returning to school said, it was hard to pump and go to school, plus "I got to the point where my nipples would sometimes bleed ... I had to just take her off altogether." Only one participant reported overcoming the pain of breastfeeding--she noted that it hurt at first, but she got used to it, and was continuing to breastfeed at seven months.

#### Difficulty latching on

Five participants encountered difficulty establishing breastfeeding and getting the baby to latch on. Three teens attributed their infants' struggles in latching on to the fact that their babies were born "small" or low birth weight. Two participants believed that their babies had trouble breastfeeding because they had been given a pacifier and formula by hospital staff before breastfeeding.

#### Concern about insufficient milk

Six participants stated that their milk "wasn't enough" or "ran out." Early struggles with their milk supply and difficulty getting the babies to accept the breast often led participants to use the breast pump or formula because, in the words of one teen, the baby "had to get fed." As one participant explained, her baby girl "would get like mad all of a sudden because she couldn't get [any milk] and just cry a lot ... so I would have to just give her a bottle" (Rural Hispanic teen breastfeeding some, but mostly giving formula at interview one month postpartum). Two participants acknowledged that they did not breastfeed as often as they should have, because they did not like doing it, and their milk supply diminished as a result.

#### Returning to school

Four participants stopped breastfeeding upon returning to school. As one participant explained, the logistical difficulties of combining school and breastfeeding were overwhelming:

I quit once I went back to school because there was like no way I could do it. That would have been really hard ... I mean, because I would have to like go pump like every two to three hours, and I just couldn't do that [Rural White teen, initially tried to breastfeed but it did not work so she pumped for two to three months and then ceased giving breast milk].

Only three participants continued to give breast milk after returning to school, and all commented how challenging it was. Specific problems included lack of sleep, breast leakage, and the difficulty and frequency of pumping.

### Influences on initiation and continuation

The only question in the PRAMS data about influences on breastfeeding was whether a doctor, nurse, or other health care worker had talked about breastfeeding during a prenatal visit. Nearly all teens (94%) reported that such a conversation had occurred (Table 3); however, it was less commonly reported among Hispanic teens (84%) than among Black (95%) or White (96%) teens.

In our qualitative interviews we assessed a broader range of influences in relation to both initiation and continuation of breastfeeding, including health care professionals, family, peers, and partners (Table 4).

#### Health care professionals

Similar to the quantitative respondents, many qualitative participants reported that their prenatal care providers talked to them about breastfeeding. Nearly all of the teens mentioned hearing that breastfeeding was good for the health and development of the baby, whether they breastfed or not. Very few participants mentioned any reasons for breastfeeding other than the baby's health and development. Only two participants reported that their prenatal care providers did not encourage them to breastfeed. One of them, a rural White teen who never breastfed, said "Through my whole pregnancy, they asked was I going to bottle or breastfeed. I wasn't sure, but none of them ... tried to encourage either one."

Many participants also obtained information about breastfeeding at the hospital after delivery. Some received hands-on assistance to show them how to hold the baby for feeding, including two teens who reported receiving help from lactation consultants. The majority of participants participated in the Special Supplemental Nutrition Program for Women, Infants and Children (WIC), which provides food and nutrition education to low-income pregnant and postpartum women, and to infants and children up to age five at nutritional risk. A few participants mentioned receiving a pump from WIC. Additional professional sources of breastfeeding information and encouragement mentioned by a few participants were a video from the WIC program, a social worker, an adolescent parenting program, and a breastfeeding class at the hospital.

The extent to which the information provided by health care professionals influenced participants' decisions to breastfeed varied. At least one participant (an urban African American participant who tried breastfeeding one time and then stopped) was resistant. She said: "The doctor said something about kids who get breastfed be smarter than the ones who don't, but I think if he was supposed to be smart, it don't matter whether I breastfeed or not." For two participants that had not previously planned to breastfeed, however, the information and encouragement they received at the hospital after delivery convinced them to try it. One rural White teen recounted that she had not planned to breastfeed because her mother had not. As she explained, "I just thought bottles because my mom, she always bottled all hers." After the baby was born, however, the hospital staff convinced her to try, and at six weeks postpartum, she was continuing to breastfeed.

Participants who attached more importance to the health and developmental benefits of breastfeeding generally persevered in breastfeeding longer than those who did not. Those who breastfed longer expressed more ownership in their statements on the benefits of breastfeeding, saying for example, "I wanted my baby to get nutrients," or as one rural African American teen who breastfeed for six months stated, "I felt that [breastfeeding] was very important because that first milk is something that can help jumpstart your baby's brain." In contrast, those who did not breastfeed as long more often described what others said about the benefits without owning the benefits themselves, such as "they say it is healthy for the baby" or "the doctors say it is better than formula".

Professional assistance with breastfeeding after hospital discharge was uncommon. Few participants mentioned discussing their breastfeeding problems with any professionals, and most of those who did reported that the responses they got were not helpful. For example, a rural Hispanic teen described how a home visitor from the health department gave her papers and magazines about breastfeeding but did not address her specific problems with nipple pain and perceived low milk supply. A few participants received pumps from the WIC program, but one teen commented that no one showed her how to use it; after pumping for one week, she stopped because her nipples cracked and bled. Another participant was advised to stop breastfeeding by her medical provider because of medication she was prescribed for the flu. A rural Hispanic teen who was trying to breastfeed at her interview six days postpartum said, "I actually told my pediatrician [about difficulties breastfeeding] and they said that, you know, sometimes babies want it and sometimes they just don't." One rural White participant who was continuing to breastfeed at her interview six weeks postpartum received assistance that she perceived to be helpful, but that was not necessarily in line with best practices. She had been worried about her milk supply, so she called the Women's Health Center and they reassured her that it was normal and gave her a pill to increase her supply, rather than advising her to increase the frequency of feedings. Despite contact with health care and social service staff during the first few weeks postpartum, in most cases teens did not receive support that facilitated continued breastfeeding.

#### Family

Fourteen out of 22 participants reported that their mothers or other women in their family had breastfed and encouraged them to breastfeed, telling them it was "healthy for the baby." For some participants, family modeling of and support for breastfeeding had a major impact on their decisions to initiate. As one rural White teen who initially tried to breastfeed but it did not work so she pumped for two to three months and then ceased giving breast milk recounted, "My sister breastfed all three of her kids as long as she could, until they were like a year old. So seeing her do that, I just tried to do it."

Strong family support for breastfeeding was most common among the Hispanic participants and least common among the African American teens. Although a few African American participants said that other women in their families had breastfed and everybody was supportive, this was not the case for most. As one urban African American participant who did not breastfeed recounted, her mother and grandmother had not breastfed, and they had no opinion about whether she should breastfeed--they just told her, '"Whatever you want to do.' And I just didn't want to do breastfeeding, so I fed him the bottles and he took the milk real good."

A commonality among adolescents who breastfed for longer periods (two months or more) was that all of them reported multiple female family members who had breastfed and encouraged them to do so. However, strong family support did not always ensure longer breastfeeding. For some participants, family support was insufficient to help them overcome the barriers they encountered and they discontinued within a few days or weeks after delivery. Although female family members offered moral support, they were not able to provide the technical guidance that many of the teens needed to overcome the difficulties they encountered. For example, one participant had difficulty getting her baby to latch, and her mother just encouraged her to "keep on trying."

#### Peers and partners

There was a general impression, even among qualitative participants who did breastfeed, that breastfeeding was uncommon among teens. A few participants mentioned that none of their friends had breastfed. For African American and White adolescents, a lack of breastfeeding and negative experiences among their peers served as influential barriers to breastfeeding. Peers were not an influence mentioned by Hispanic adolescents, however. Of the five teens who never breastfed, three based their decisions on stories of their friends' and similar-aged relatives' negative experiences, including pain and logistical challenges. As a rural White teen who did not breastfeed recounted, "During my pregnancy I did [think about breastfeeding] but ... my cousin breastfed, and she said that she missed a feeding and her breast got clogged, and that was ... a scary thing to hear. So it changed my mind." A rural African American who did not breastfeed said, "[My friend] had to take her breast pump to school and pump while she was at school. I was like, 'Naw, I can't do that.'" Interestingly, no participants mentioned their partners when discussing their decision to breastfeed.

## Discussion

Our combined quantitative and qualitative approach enabled us to both quantify breastfeeding practices among young adolescent mothers, and to explore in depth the factors that promote and inhibit breastfeeding. To our knowledge, no previous published reports using population-based data have described breastfeeding practices specifically among adolescents (ages 17 and younger). We found that the proportion of teens in North Carolina that was motivated to try breastfeeding (52%) was quite similar to national data for teens 19 and younger in 2004 to 2008 participating in the National Immunization Survey (53.0%: Confidence Interval (CI) 46.1, 59.9) [1]. However, the duration of breastfeeding was much shorter: Only 28% of teens in North Carolina continued to breastfeed four weeks after delivery, whereas among teens 19 and younger nationally, 19.3% (CI: 13.8, 24.8) were breastfeeding *six months *after delivery [1]. Furthermore, rates of exclusive breastfeeding were much lower among teen mothers in North Carolina. Less than 17% reported exclusively breastfeeding through four weeks postpartum, whereas nationally the same proportion (17%, CI: 10.3, 23.3) of mothers 19 and younger reported exclusive breastfeeding through three months [13].

We also observed distinct patterns across racial-ethnic groups. Hispanic teens had the highest rates of breastfeeding initiation and longest duration, followed by White and African American adolescents. Among adult mothers nationally, racial-ethnic patterns are similar, but differences are less pronounced [1]. The exceptionally high breastfeeding rates among Hispanic teens in our study may relate to the fact that nearly 60% of Hispanics in North Carolina are immigrants [14], and less-acculturated immigrants have higher breastfeeding initiation rates and breastfeed for longer durations than U.S.-born women [15,16].

The observed differences by race/ethnicity in the quantitative data reflect the social and cultural norms on breastfeeding that emerged in the qualitative study. The impact of teens' social of contexts was most evident in the decision to initiate breastfeeding. In our qualitative sample, more Hispanics than either Whites or Blacks had a family history of breastfeeding and reported the most encouragement from family. African American teens were less able to articulate exact reasons for not breastfeeding, and their discourses reflected family norms in which formula feeding was a more accepted form of infant feeding [5]. Moreover, negative stories of breastfeeding experiences were pervasive among White and Black participants' peer groups. Despite previous studies pointing to the role the baby's father plays in the decision to breastfeed and providing support to breastfeeding teens [5,8], adolescents in our study did not report receiving information or support from partners.

Given that family support was very influential in teens' decisions to breastfeed in this study and in previous research, measures to increase the involvement of teens' mothers in breastfeeding counseling and education may enhance teens' motivation to breastfeed. Peer counseling by breastfeeding adolescents to share positive experiences and to acknowledge difficulties in advance so that teens are prepared for the commitment that breastfeeding takes--possibly through online groups or social networking sites--may also be a promising strategy warranting more research.

Although the perceived health benefits of breastfeeding and family encouragement motivated many teens to try breastfeeding, they were not necessarily enough to keep them motivated when they encountered difficulty. In both the quantitative and qualitative samples, high rates of discontinuation occurred within the first month postpartum. Reasons for cessation in the first four weeks were largely due to lactation difficulties including sore nipples, perceiving that they were not producing enough milk or that the baby was not satisfied, and trouble getting the baby to latch. These reasons are similar to previously published reports of PRAMS data among women of all ages [17] and data from the Infant Feeding Practices Study II among adult mothers [18]. This suggests that supplementary professional support in the early postpartum period to address these technical challenges, which has been successful in increasing breastfeeding duration among adult women [19], may be warranted for teens.

However, few participants in our qualitative study reported receiving professional support for breastfeeding after hospital discharge and none received any hands-on technical support. Strategies that may be helpful include more hands-on tailored support through the early postpartum period, both in the hospital and at home to provide assistance on specific problems that adolescents encounter. These include ways to avoid and treat nipple pain, help the baby to latch properly, stimulate and maintain the milk supply, and use the breast pump. Counseling that emphasizes that breastfeeding is a learning process [20] and provides adequate information about the frequency and duration of breastfeeding and how to determine whether their infant is getting enough milk may also be beneficial. This concrete help, along with reassurance that the problems are commonplace and that assistance is frequently needed to overcome the challenges among mothers of all ages, may help build teens' confidence and help prevent early cessation.

School was another major barrier to breastfeeding identified by teens in both the quantitative and qualitative studies. The prevailing view was that breastfeeding was not compatible with school. In contrast to our findings, a recent study documented that with adequate support from school and the provision of onsite daycare, teens successfully continued breastfeeding upon return to school [8]. A large portion of teens in our qualitative study received homebound education services from their schools during the first weeks postpartum. This period may provide an opportunity to communicate to teens that breastfeeding for a short period of time is better than not breastfeeding at all, and to help teens prepare for the transition back to school. Once teens return to school, flexibility in their schedule, a private place for expressing milk that is not a restroom, the use of breast pads to cover up leaking, and storage for expressed milk and the breast pump could help teens to succeed in both academics and breastfeeding. As part of the Affordable Care Act enacted in 2010 in the United States, the Fair Labor Standards Act was recently amended to require employers to provide break time and a private place to express milk while at work [21]. Advocacy efforts are needed for teen mothers to receive similar support in schools. Schools could also support breastfeeding among teen mothers by integrating breastfeeding education into school health programs [22].

Future research should investigate what is the best way and who is best positioned to provide concrete help to breastfeeding teens within the first week postpartum [23]. Since early breastfeeding experiences affect new mothers' confidence in their ability to breastfeed and their motivation to continue [20], it is important that teens receive support within the first two or three days following hospital discharge. Given previous research, and our findings that teens may not feel as comfortable to ask for help from health care providers [7,8], further research should also explore whether teens might benefit from being offered extra support regardless of whether or not they are experiencing difficulty. This might consist of a home visit, or an outpatient visit as a second option, that is scheduled prior to hospital discharge [24]. Home visits could be provided by a lactation consultant, certified lactation counselor, or staff from an adolescent parenting program trained in breastfeeding.

Several limitations should be considered when interpreting the findings of this study. The sample size for the quantitative data is relatively small, particularly for sub-groups such as for Hispanic teens, so results should be interpreted with some caution. Although survey data are weighted for nonresponse to reduce bias, maternal age less than 20 years is a predictor of nonresponse in the North Carolina PRAMS, as well as African American or Hispanic background [25]. Given this potential bias, teens participating in PRAMS may be more highly selected and may be more motivated to breastfeed than those who did not respond. Thus our prevalence estimates may be higher than in the general population. In both the quantitative and qualitative study components, respondents provided information about infant feeding experiences retrospectively which may be subject to recall bias. The majority of participants in both the quantitative and qualitative studies either did not breastfeed or stopped within the first month postpartum; information on infant feeding was obtained from PRAMS participants between two and seven months postpartum and from qualitative participants between one and 18 months postpartum.

In our qualitative study, we interviewed a non-representative sample of teen mothers. By recruiting mothers through organizations serving adolescents, we may have missed the most marginalized teens. However, our multifaceted recruitment approach enabled us to interview adolescents who were both in and out of school, from rural and urban areas in North Carolina, with varying levels of programmatic and clinical support, and may be particularly relevant for programs and service providers in understanding the needs of teens they serve.

## Conclusion

This mixed-methods study extends earlier findings on adolescent breastfeeding and the factors that promote and inhibit breastfeeding throughout the first year postpartum. We found that adolescent mothers were hearing the message that breastfeeding was the ideal form of infant feeding and over half were motivated to try. However, they encountered many barriers that prohibited them from continuing. For most teens, the hassle of breastfeeding and problems encountered without the practical support to overcome them were so great that they did not persist for long. Our findings suggest that prenatal education and counseling for teens may be more effective if it also included female family members and targeted teens' peer networks. Provision of an extra home visit or outpatient visit for teens within the first few days following hospital discharge consisting of hands-on technical support could enhance their ability to breastfeed. Additionally, more assistance with the transition back to school and advocacy to make schools more compatible with breastfeeding have the potential to help teens who desire to breastfeed to successfully continue. These interventions warrant further research to test their effectiveness among adolescents.

## Competing interests

The authors declare that they have no competing interests.

## Authors' contributions

CT performed the qualitative analysis and drafted the manuscript. EW designed and managed the study, developed the semistructured interview guide, collected the qualitative data, participated in the interpretation of the data, and drafted and revised the manuscript. GS collected the qualitative data, participated in the interpretation of the data, and revised the manuscript. All authors read and approved the final manuscript.
